# Silvicolous on a Small Scale: Possibilities and Limitations of Habitat Suitability Models for Small, Elusive Mammals in Conservation Management and Landscape Planning

**DOI:** 10.1371/journal.pone.0120562

**Published:** 2015-03-17

**Authors:** Nina I. Becker, Jorge A. Encarnação

**Affiliations:** Mammalian Ecology Group, Department of Animal Ecology and Systematics, Justus-Liebig-University of Giessen, Giessen, Germany; Università degli Studi di Napoli Federico II, ITALY

## Abstract

Species distribution and endangerment can be assessed by habitat-suitability modelling. This study addresses methodical aspects of habitat suitability modelling and includes an application example in actual species conservation and landscape planning. Models using species presence-absence data are preferable to presence-only models. In contrast to species presence data, absences are rarely recorded. Therefore, many studies generate pseudo-absence data for modelling. However, in this study model quality was higher with null samples collected in the field. Next to species data the choice of landscape data is crucial for suitability modelling. Landscape data with high resolution and ecological relevance for the study species improve model reliability and quality for small elusive mammals like *Muscardinus avellanarius*. For large scale assessment of species distribution, models with low-detailed data are sufficient. For regional site-specific conservation issues like a conflict-free site for new wind turbines, high-detailed regional models are needed. Even though the overlap with optimally suitable habitat for *M*. *avellanarius* was low, the installation of wind plants can pose a threat due to habitat loss and fragmentation. To conclude, modellers should clearly state the purpose of their models and choose the according level of detail for species and environmental data.

## Introduction

Habitat-suitability models are used to assess species distribution and endangerment [1]. The resulting suitability maps permit a scientific statement on the area of suitable habitats of single species and their potential distribution in the landscape [1]. Model performance depends on model type and implementation but especially on quantity and quality of species and landscape data [2, 3].

It is preferable to model with species data that contain confirmed presences and absences [4–6]. However, high quality species absence data are scarce as studies usually focus on species presences [5] and absences are much more difficult to verify [7]. There are several ways to be still able to model with presence/absence data. Pseudo-absences can be randomly generated [4]. However, these pseudo-absences might strongly influence final model quality as they might be e.g. located in suitable habitat [4, 8]. Another possibility to obtain absence data are voluntary surveyed data sets. These frequently contain null samples where no indication for a species was found. These are of value when standards for obtaining null samples have been clearly defined [9]. While this is no guarantee of species absences [7] it might be an improvement over randomly generated absences. In many European countries (e.g. Poland, Denmark, France, England), voluntarily surveyed data are already integrated into national monitoring programmes [10–12]. In Germany, voluntary efforts are also increasingly being used, as exemplified by the beaver survey in the Spessart [13]. Voluntarily surveyed data often provide a major basis for species distributions and might offer the possibility to generate accurate and reliable suitability maps.

Next to high quality species presences and absences landscape data with high resolution improve model reliability and quality [14, 15]. However, detailed data with high resolution are unlikely to be available at landscape level [1, 16] and consequently have to be obtained either at great financial cost and/or with great effort. Models using low-detailed data have already been proven effective for bird species [17, 18], insects [19], and larger mammals [20, 21], but evidence remains scare for smaller, more elusive mammals [22, 23].

Many of these small and elusive mammals are legally protected and in the Annexes of the European Council Directive 92/43/EEC on the Conservation of natural habitats and of wild fauna and flora (Fauna-Flora-Habitats Directive) [24, 25]. In addition to this legal protection, the Member States of the European Union are obliged to record species condition and to find measures to contribute to their conservation. However, estimation of their distribution and endangerment is methodically difficult due to their concealed and nocturnal way of life. Reliable and high quality suitability models can help to improve our understanding of species distributions. This study addresses the suitability of different modelling performances for landscape planning and assessment using a current example from Germany: the energy turnaround.

Since the amendment of the German Building Code in August 1997 wind turbines are privileged objectives. To avoid conflicts with public interest wind turbines are mostly erected in outlying areas like forests. However, these areas might be suitable habitat for legally protected species. To minimize this conflict the Building Code incorporates a plan reservation for the spatial control of wind turbines. Precedence areas are identified and the remaining landscape areas represent exclusion zones for regionally significant wind turbines. The installation and operation of wind plants can, therefore, pose a threat to small and elusive mammals by habitat loss and habitat fragmentation due to the construction of turbines and supply channels [26–28].

The achieved aims of this study were to identify 1) whether models with null samples predict better than those with randomly generated pseudo-absences and 2) how the inclusion of landscape detail influenced model accuracy. These insights were then used to assess 3) the potential habitat loss caused by precedence areas of wind turbines for a protected small, elusive mammal.

## Materials and Methods

### Study area

**Fig 1:**
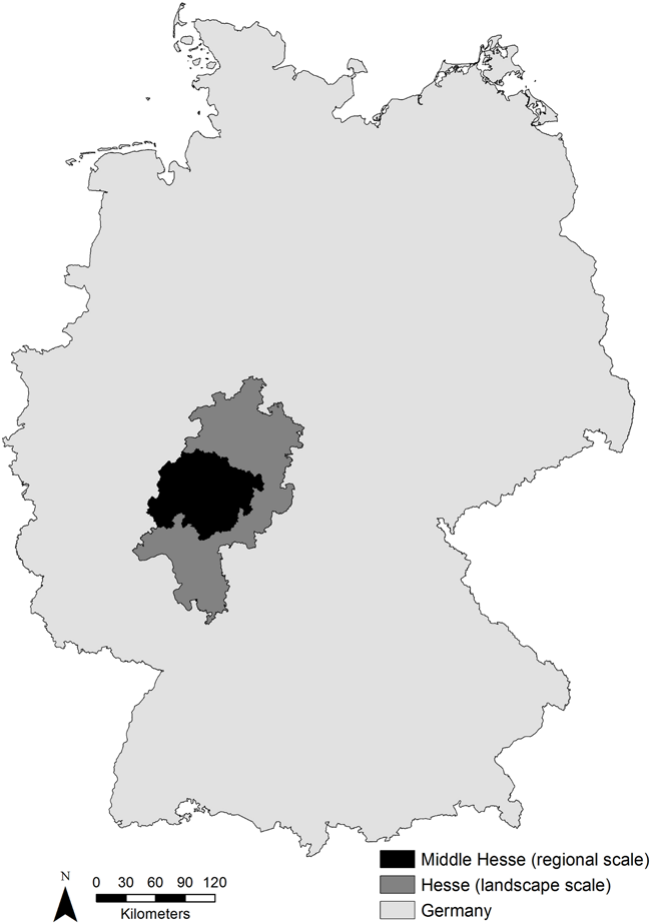
Regional (Middle Hesse) and landscape (Hesse) study area in Germany.

The study was conducted in Hesse, Germany. The landscape scale in this study was represented by the whole of Hesse. It is located in central Germany with an area of 21119.2 km^2^ (Fig. 1). The state is characterized by the Central German Uplands (up to 950 m asl) and basin landscape (up to 200 m asl). Other federal states have an average of 30% forest area while Hesse is more arboreous with 41% (8732.4 km^2^) forest cover. The regional scale of this study was Middle Hesse (Fig. 1). It lies at the centre of Hesse with an area of 6644.4 km^2^ (31% of Hesse) and a forest cover of 39% (2611.3 km^2^). Therefore, Middle Hesse is representative of the state of Hesse as a whole. The field permit was granted by the Regional Council Giessen.

### Study species

We chose the common dormouse (*Muscardinus avellanarius*) as model species for small, elusive mammals. *Muscardinus avellanarius* has been assigned ‘least concern’ status by the IUCN Red List of Threatened Species and is listed in annex IV of the Habitats Directive [24, 25]. It occurs in small population densities and requires well networked woodland, as it avoids open land [29, 30]. It is highly vulnerable to habitat fragmentation as it moves among branches in 2–5 m height avoiding the forest floor [30, 31]. Thus a supply channel for infrastructure e.g. wind parks in forests can already be a crossing barrier for *M*. *avellanarius* [31]. In the Red List for Germany, a decline and an endangerment of unknown proportions is assumed for *Muscardinus avellanarius* [32]. A decline is also to be assumed in Hesse, on the basis of the results of a nationwide monitoring programme conducted since 2006. However, a realistic estimation of the conservation status of the dormouse on the basis of current officially surveyed data is difficult [33].

### Species records as presence data

The records for the dormouse originate from three data sources (1–3) for Hesse, Germany (Table 1). The official data set (1) of *M*. *avellanarius* was provided by the central, state-wide database of Hesse (Hessen Forst FENA) and was supplemented by own records (2). The official data set (1) was collected based on the standard given by the Hessen Forst FENA (Forest Inventory and Nature Conservation Agency of Hesse) [34]. In 35 monitoring plots over Hesse 50 nest boxes were installed in 4 rows with a distance of 50 m to each other (approx. 16 ha). Since 2006 boxes were controlled for nests or individuals of *M*. *avellanarius* in June and September. Own data (2) were collected since 2011 using nest tubes (n = 50, 25 x 6.5 cm) that were distributed in up to 11 forest plots in a grid of 120 x 120 m. They were installed below branches with the opening towards the tree trunks in a height of 1–2 m. To prevent flooding of tubes during rain tubes were installed slightly slanting. Tubes were controlled monthly from February to August for nests or individuals of *M*. *avellanarius*. On the initiative of the Naturschutzbund Deutschland Landesverband Hessen e. V. (Hesse branch of the German Society for Nature Conservation, NABU Hessen), the Great Nut Hunt was conducted throughout Hesse since 2005. As this was a voluntary initiative these data represent the voluntarily surveyed data set (3). Within this context, hazelnuts were collected by kindergarten and school classes, as well as local conservation associations, in order to look for species-specific bite marks of *M*. *avellanarius* [9]. Hazelnuts were collected in areas of 10 x 10 m around hazel bushes (*Corylus avellana*) for 20 min [9]. The whole search duration was 2h and presence detection using this method is 80% even though it is strongly biased by the occurrence of hazel bushes [9]. Hazel nuts thought to be opened by *M*. *avellanarius* were send to experts of the NABU Hessen for confirmation (http://hessen.nabu.de/projekte/nussjagd). These data are high quality presence data [35]. To keep the data up-to-date only presences from the years 2007 to 2013 were used (temporal verification). Only points with adequate (±50 m) spatial accuracy were selected. To balance over- and underrepresentation of areas in modelling we only used presence points with a minimum distance of 200 m to each other (spatial verification) [30, 36]. A lower value would lead to spatial clustering. A higher value would result in an underrepresentation of densely occupied and, therefore, most probably optimal habitat. Presence points were mostly in forests (56.6%) or in green areas like gardens or parks (24.1%). Some presences were found in agricultural areas like small groves or hedges (19.3%). After spatial and temporal data verification the official data set thus contained 190 and the voluntary data set 167 explicit presence points. The processed official and voluntary data sets were combined (357 points) for the analysis on a landscape scale (combined model). For the models on a regional scale with detailed forest characteristics only points in forests where detailed data were available were used. This reduced the combined data set to 52 presences.

**Table 1:** Summary of BRTs.

Model	Scale	Detail	Records	Cv AUC	TSS	PCC	MRD	TN
**Official**	Landscape	Low	190/190	0.76 ± 0.01	0.67	0.84 ± 0.02	0.76	5100
**Voluntary**	Landscape	Low	167/167	0.82 ± 0.03	0.72	0.86 ± 0.02	0.58	6700
**Combined**	Landscape	Low	357/357	0.83 ± 0.01	0.73	0.87 ± 0.01	0.55	2300
**Combined**	Regional	Low	52/52	0.80 ± 0.04	0.67	0.84 ± 0.04	0.73	2450
**Combined**	Regional	High	52/52	0.82 ± 0.04	0.66	0.81 ± 0.04	0.67	2600

Scale, detail (included detail of landscape data), records (presence/absence), cv AUC ± SE, True Skill Statistic (TSS), Percent Correctly Classified (PCC), mean residual deviance (MRD), and optimal tree number (TN). Mean total deviance was 1.39. Absences in the official model were pseudo-absences.

### Null samples and random points as pseudo-absence data

Positive species records are generally at the centre of surveys and thus are recorded. Data on species absences, however, are rare but important as presence-absence models are preferable to presence-only models [37, 38]. The voluntary data set contained null samples. These null samples were interpreted as evidence of absence, since no nuts were found that were opened by *M*. *avellanarius* in these areas. The search effort was the same as for presence points (20 min per hazel bush, 10 x 10 m squares, 2h overall). As both presence and absence data were sampled in the same manner they had the same sampling bias (http://hessen.nabu.de/projekte/nussjagd). Absence probability using this method in 5 squares (10 x 10 m) in one area is 90% [9]. The occurrence of dormice cannot be completely ruled out but these absence data are of the highest quality obtained to date. Absence points were selected by date (2007–2013), spatial accuracy (±50 m), distance to presence points (min. 800 m), and to other absence points (min. 200 m). For the voluntary (167 points) and the combined (357 points) data set a subsample equal to the number of presence points were randomly chosen to obtain balanced samples of presence and absence data. For the detailed models including forest characteristics 52 absence points in forests were randomly selected. Randomness was assured by using ArcGIS Desktop (ArcMap Version 9.3.1, ESRI Inc., Redlands) and Hawth’s Analysis Tools v3.27 [39].

The official data set contained only presence records (n = 190). To balance presence and absence data, the same number of pseudo-absence points was generated using Hawth’s Analysis Tools v3.27 [39] completely at random [4]. This is an often-used method [40], even though it might strongly influence the quality of the final model [8]. To increase absence probability points had a minimum distance of 800 m to presence points. To prevent a clustered distribution of the pseudo-absence points we enforced a minimum distance of 200 m between all points.

### Environmental variables

Environmental variables were chosen based on the ecology of the study species [29, 41–43]. In the landscape models freely available data of climate [44], elevation (German Federal Agency for Cartography and Geodesy), and landscape (Official Topographical Cartographic Information System (ATKIS), Hessian State Office of Land Management and Geological Information) were used. For 20840.7 km^2^ of Hesse data were available which comprise the landscape study area. The costly detailed forest inventory data included in the regional model held information on forest structure, age, and tree species composition (Hessen Forst FENA). Data were costly in terms of financial cost but even more so in time spent acquiring and preparing data for usage. Of an overall of 2611.3 km^2^ of forest in Middle Hesse we obtained inventory data for 1699.2 km^2^ which is the regional study area. All landscape data were rastered at a resolution of 25 x 25 m. To account for spatial inaccuracy in point and continuous landscape data focal statistics were calculated over a neighbourhood of 100 m (ArcMap Version 10.1, ESRI Inc., Redlands). For categorized landscape data the percentage of occurrence and the diversity (Interspersion and Juxtaposition Index (IJI), Shannon's Diversity Index (SHDI)) around 100 m of the point (Fragstats 4.1) was considered in the analysis.

### Modelling

In this study five boosted regression tree (BRT) models were built [45] (Table 1). On the landscape level the predictive performance using the pseudo-absences and the null samples of the voluntary data set were compared. Then the datasets were combined in the overall landscape model to assess the influence of sample size on model quality. To evaluate the influence of detailed forest inventory data on model performance two models on a regional scale were fitted. Models were implemented in the statistical program R (R Development Core Team, [46]) with the gbm libraries [47] and the modifications of gbm.step provided by Elith et al. [48]. The main parameters of a BRT are model complexity (learning rate), stochasticity (bag fraction), number of trees contributing to the model, and fitted interactions (tree complexity) [48]. They were determined by 10-fold cross-validation (cv) and the best model was chosen when deviance reduction was greatest [48, 49]. For all models learning rate was set to 0.001, bag fraction to 0.5 and tree complexity to 5. All environmental variables were used for all models while the detailed models included also the forest inventory data. Best environmental predictors were chosen using the function gbm.simplify [48]. Model performance was evaluated by area under curve for cross-validation data (AUC ≥ 0.90: excellent; 0.90 > AUC ≥ 0.80: good; 0.80 > AUC ≥ 0.70: fair discrimination ability [50]), deviance reduction (the lower the value the better the model), the True Skill Statistic [51] (TSS ≥ 0.75: excellent; 0.75 > TSS ≥ 0.40: good; TSS < 0.40: poor discrimination ability [52]), and the percent correctly classified (PCC). The cv AUC is usually much lower than the AUC but a much better predictor of model performance [2, 53].

Habitat suitability models were spatially predicted using the predict version in the R package 'raster'. The result was a habitat suitability map with the suitability ranging from 0 (low suitability) to 1 (high suitability). We categorized suitability based on Jenks natural breaks classification method (suitability: optimal ≥ 0.9; 0.9 > moderate ≥ 0.5; low < 0.5) (ArcMap Version 10.1, ESRI Inc., Redlands). This data mining method reduces the variance within categories and maximizes the variance between categories.

### Application example: Wind energy

**Fig 2:**
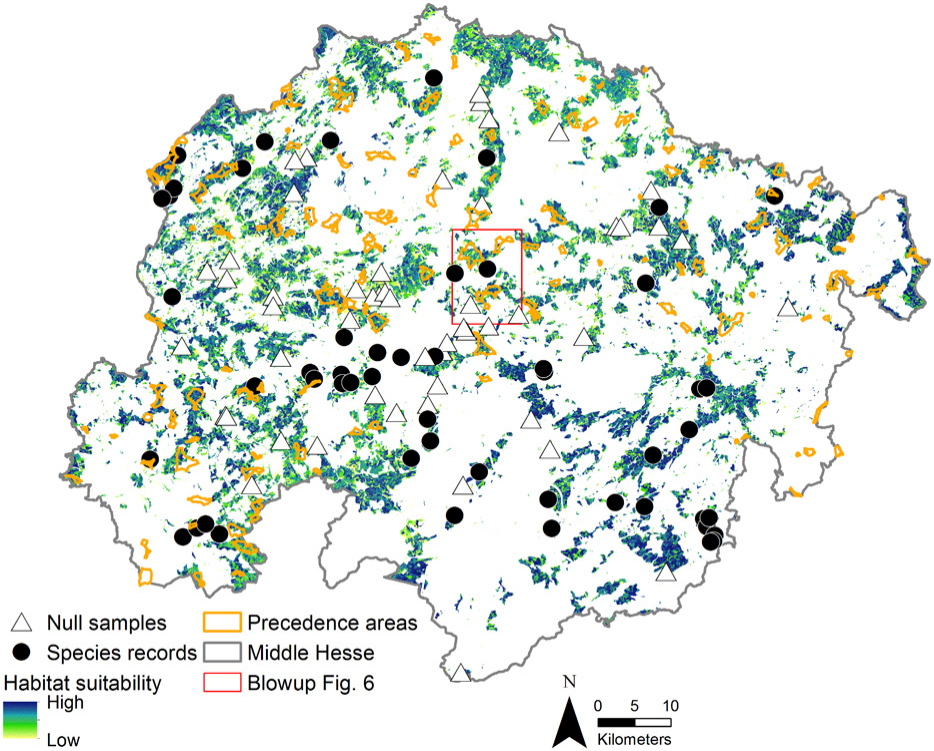
Habitat suitability for *M*. *avellanarius* in Middle Hesse with precedence areas for wind turbines. The habitat suitability was based on the high-detailed regional model.

Precedence areas for wind turbines are areas where turbines can be built and the remaining landscape areas represent exclusion zones. For Middle Hesse 134 precedence areas were designated covering an area of 164.7 km^2^ (2.5% of Middle Hesse, Regional Plan for Middle Hesse 2012). They are mainly located in the northern and western part of Middle Hesse in elevations of 144–674 m (371 ± 89 m) with high wind potential. Area size ranges from 0.1 km^2^ to 8.1 km^2^ and 0.7% is located in urban areas, 15.2% in agricultural landscapes and the vast majority with 84.2% in forests (Fig. 2). The impacts of wind turbines on *Muscardinus avellanarius* are both short-term (e.g. disturbance during construction) and long term (e.g. habitat modification, loss or fragmentation) [28, 54]. To assess the possible long-term impact on *M*. *avellanarius* the overlap between moderate and optimal habitat for *M*. *avellanarius* as spatially predicted by the habitat suitability models and precedence areas (Regional Plan for Middle Hesse 2012) for new wind turbines was calculated by computing the geometric intersection of areas.

## Results

### Better model performance with null samples and higher sample size

The model based on the official data set for Hesse (species records, n = 190) in combination with random pseudo-absence points (n = 190), had a cv AUC of 0.76 ± 0.01 standard error (SE) and a TSS of 0.67. Mean total deviance was 1.39, mean residual deviance 0.76 and cv deviance 1.14 ± 0.03 SE. Optimal tree number for the model was reached at 5100 trees (Table 1).

The voluntary data set with presence data (species records, n = 167) and absence data (null samples, n = 167) for Hesse produced a cv AUC of 0.82 ± 0.03 SE and a TSS of 0.72. The mean total deviance (1.39) was markedly reduced to 0.58 and the cv deviance was with 1.04 ± 0.06 SE lower than for the official dataset. The number of trees included in the model was 6700 (Table 1).

**Fig 3:**
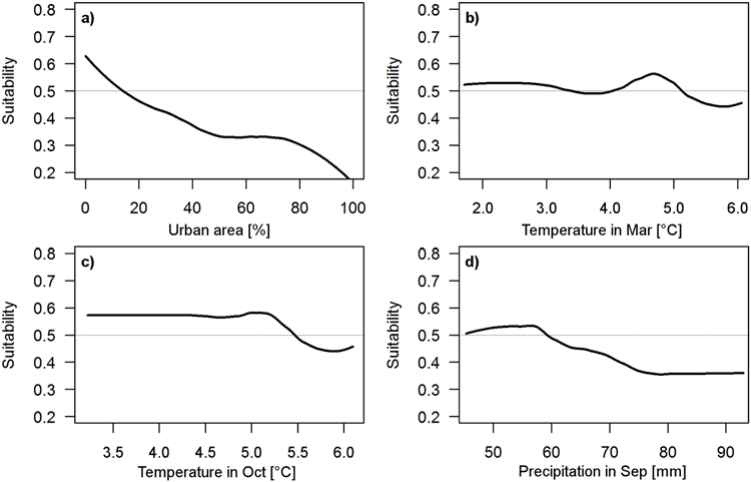
Response curves of the combined landscape model. Optimal habitat for *M*. *avellanarius* has a low percentage of urban area (a), ideally 4–5°C in March (b), cold ambient temperatures in October (c) and low precipitation in September (d).

**Fig 4:**
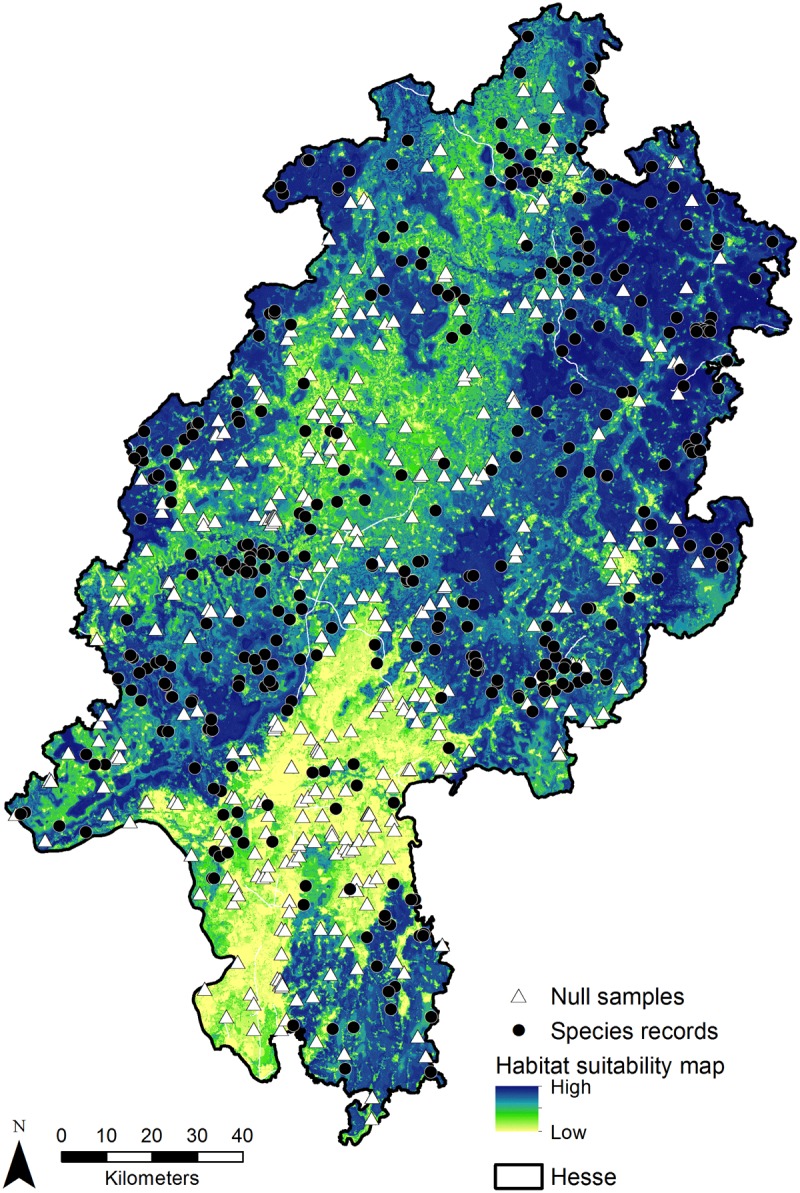
Habitat suitability for *M*. *avellanarius* in Hesse. Darker colours indicate more suitable habitat, triangles null samples collected in the field, and circles species presence data.

Combination of the two data sets (combined species records: n = 357, null samples: n = 357) led to a better model compared with the individual models, with a cv AUC of 0.83 ± 0.01 SE and TSS of 0.73, accompanied by a high mean deviance reduction (1.39 to 0.55) and cv deviance of 1.02 ± 0.03 SE. Optimal tree number for the model was 2300 (Table 1). The model consisted of the ten most influential predictors: percentage of urban area (Fig. 3a) and landscape diversity (IJI) 100 m around the data point, ambient temperatures in March (Fig. 3b), April, October (Fig. 3c), and December, ambient temperature seasonality, and precipitation in June and September (Fig. 3d). Based on the combined model a map of the potential distribution at landscape scale (Hesse) was visualised (Fig. 4). This map can be used to differentiate between habitats with low (model prediction < 0.5), moderate (model prediction ≥ 0.5), or optimal (model prediction ≥ 0.9) suitability or occurrence probability of *M*. *avellanarius* (Table 2).

**Table 2:** Areas and percentages for all five BRT models.

	Optimal suitability	Moderate suitability	Low suitability
	[km^2^/%]	[km^2^/%]	[km^2^/%]
**Official landscape model**	2822.7/13.5	8346.7/40.0	9671.3/46.5
**Voluntary landscape model**	4235.3/20.3	8379.3/40.2	8226.1/39.5
**Combined landscape model**	8283.9/39.7	8184.1/39.3	4372.7/21.0
**Low-detailed regional model**	687.9/40.5	906.2/53.3	105.1/6.2
**High-detailed regional model**	592.1/34.8	870.2/51.2	236.9/13.9

Percentages are calculated in relation to the respective reference area (landscape or regional study area).

### High-detailed landscape data produced more realistic models

**Fig 5:**
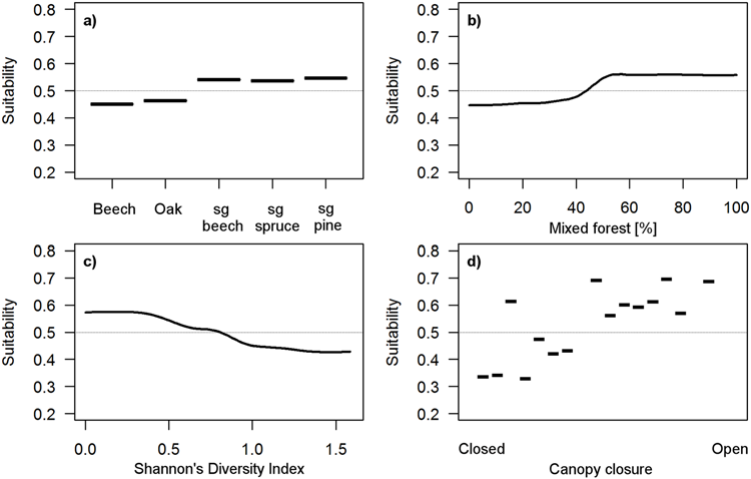
Response curves of the high-detailed regional model. Optimal habitat for *M*. *avellanarius* has a high tree species diversity (sg: species group) (a), a high proportion of mixed forest (b), a low landscape diversity i.e. only forest areas (c), and a more open canopy (d).

Two regional models were calculated with each 52 presences and absences. In one detailed forest characteristics were included. The regional model with low detail had a cv AUC of 0.80 ± 0.04 SE and a TSS of 0.67. It reduced mean total deviance from 1.39 to 0.73 and had a cv deviance of 1.12 ± 0.09 SE. Optimal tree number for the model was reached at 2450 trees (Table 1). The model with high detail had a slightly higher cv AUC of 0.82 ± 0.04 SE and reduced mean deviance to 0.67. It had, however, a slightly lower TSS of 0.66. The cv deviance was 1.15 ± 0.07 SE. Model tree number was optimal at 2600. There was a high overlap of variables in the two models. However, the two most influential variables differed. In the low-detailed model it was urban area and precipitation in September. These two variables were moved to third and forth position in the high-detailed model as canopy cover within the forest at main and pioneer level became better predictors for habitat suitability of *M*. *avellanarius*. Forest species composition was also included in the high-detailed model (Table 3, Fig. 5a-d). The low-detailed regional model predicted 687.9 km^2^ (40.5%) of the regional study area as optimal habitat for *M*. *avellanarius*. This area was reduced to 592.1 km^2^ (34.8% of the regional study area) in the high-detailed model (Table 2). Overlap of optimal and moderately suitable areas of the two regional models was high (96%). Looking only at optimal habitats area overlap was lower (54%).

**Table 3:** Predictors for the combined landscape model and the two regional BRT models.

Model	Predictor	Relative influence [%]
**Combined landscape model**	Closed development	18.04
	Mean T_a_ in Mar	11.36
	T_a_ seasonality	10.26
	Minimal T_a_ in Oct	9.81
	Open development	9.32
	Precipitation in Sep	8.90
	Maximal T_a_ in Dec	8.55
	Minimal T_a_ in Apr	8.41
	Landscape diversity (IJI)	7.87
	Precipitation in Jun	7.48
**Low-detailed regional model**	Closed development	15.76
	Precipitation in Sep	15.24
	Landscape diversity (SDHI)	12.84
	Hedges	10.43
	Precipitation in Apr	9.09
	Mixed forest	8.49
	Minimal T_a_ in Oct	8.48
	Precipitation of Wettest Month	7.17
	Precipitation in Dec	6.34
	Precipitation in Aug	6.17
**High-detailed regional model**	Canopy cover (main layer)	22.97
	Canopy cover (pioneer layer)	22.16
	Closed development	12.70
	Precipitation in Sep	8.63
	Landscape diversity (SDHI)	8.13
	Mixed forest	6.21
	Minimal T_a_ in Oct	5.82
	Mean T_a_ of wettest quarter	5.02
	Tree species composition	4.94
	Hedges	3.42

T_a_: Ambient temperature

### Overlap of optimal habitat and wind energy precedence areas

**Fig 6:**
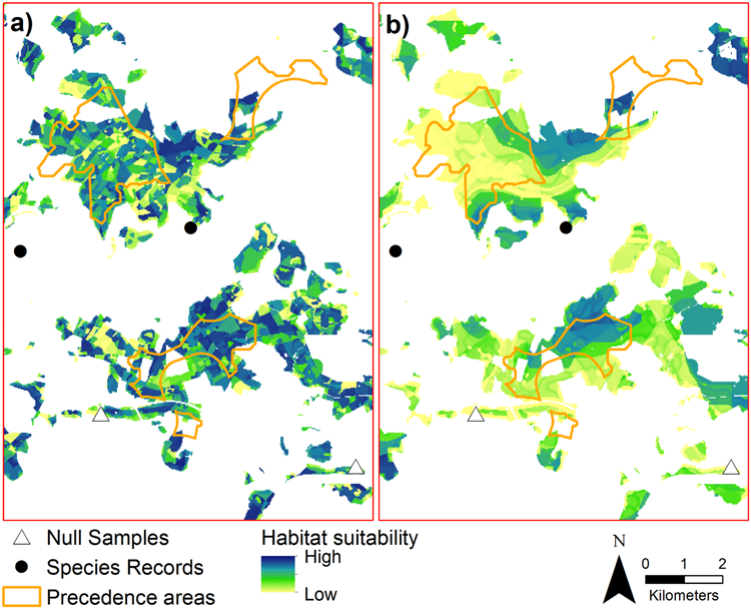
Blowup from Fig. 2 to compare the habitat suitability for *M*. *avellanarius* based on the high- (a) and low-detailed (b) regional model. Overlap of precedence areas for wind turbines and optimal habitat for *M*. *avellanarius* can lead to severe habitat loss and fragmentation.

Using the moderately suitable area of the low-detailed regional model (906.2 km^2^) as reference area 5.2% (47.2 km^2^) of suitable area was within the precedence areas. For the optimal habitats (687.9 km^2^) it was 6.2% (42.7 km^2^). Based on the high-detailed regional model 5.7% (49.6 km^2^) of moderately suitable area (870.2 km^2^) and 5.9% (34.9 km^2^) of optimally suitable area (592.1 km^2^) were within precedence areas. Precedence areas (164.7 km^2^) consisted of 28.7% / 30.1% moderately and 25.9% / 21.2% optimally suitable area as predicted by the low- / high-detailed regional model, respectively (Figs. 2 and 6).

## Discussion

### Optimal habitat for *Muscardinus avellanarius*

On a landscape level environmental and landscape variables accurately predict habitat suitability for *M*. *avellanarius*. Two landscape variables were good determinants of habitat suitability: urban area and landscape diversity. Both values should be low as *M*. *avellanarius* is a forest-dwelling species with low dispersal ability outside of forests or grooves [55, 56]. *Muscardinus avellanarius* is sensitive to ambient temperature and precipitation [57] which became apparent in the different models as many explanatory variables concern these two factors. Ambient temperature data were most important during early spring and winter suggesting that they play a role in hibernation patterns [57]. For a successful hibernation ambient temperatures should be steady and low (1–4°C [57]) as was also determined in the models of this study. Precipitation values might also be connected to hibernation patterns but also seems to be important during summer. *Muscardinus avellanarius* is sensitive to rain due to its fur characteristics and drier summers increase food availability [57]. Therefore, suitable habitats are characterized by lower precipitation values.

The importance of these variables is retained in the regional models. However, detailed forest characteristics like canopy cover or forest species composition have a predominant role in determining habitat suitability for *M*. *avellanarius*. This is to be expected as *M*. *avellanarius* is a silvicolous species and small scale data better describe resource availability like food or shelter [29, 42].

### Better model performance with null samples and higher sample size

Sampling bias in species presence data is a serious problem for species distribution modelling as in the worst case not the species distribution is modelled but sampling effort [4]. This bias has a higher impact on presence-only models than on presence-absence models which in turn makes the latter models preferable [4]. There are several types of absence data. They can be randomly generated as in this study, they can be selected with the same bias as the presence data if the bias is known [4], and they can be sampled in the field (also this study). Even though this field data cannot be considered as certain absences they have when sampled in a standardized way a higher quality than randomly generated absences as was shown in this study. However, even with these null samples a bias is introduced into the model as detection failure is influenced by sampling effort, habitat structure, accessibility of the sampling location and elusiveness of the studied species [7, 58]. Especially in voluntarily surveyed data sets bias might be considerable as sampling effort is focussed on easily accessible sites. In this study the abundance and occurrence of hazel bushes also biased voluntary data. Hazel bushes are, however, abundant and occur in most forests throughout Hesse [59] minimizing the bias on presence and absence data. Model performance was better with null samples in this study and therefore choosing even biased null samples might sometimes be preferable to generated absences. Furthermore, available data sets should be merged to increase sample size as this positively influences model accuracy [3, 60, 61]. To conclude null samples are preferable to random pseudo-absences and might be obtained by including standardized voluntarily surveyed data sets in the analysis. This in turn also increases sample size. The reduction of TSS values in the detailed models clearly showed that an increased sample size is valuable for robust models.

### High-detailed landscape data produced more realistic models

It is probably not surprising that models using high-detailed landscape data produce more realistic species distribution models. This is, however, only the case when the detailed data include ecologically valuable information for the target species [62]. In this case we included detailed forest characteristics since *M*. *avellanarius* is a forest-dwelling species [63]. Even though these data were costly in time and money, the effort was well worth as it improved the species distribution models. The most influential factors both described canopy cover. A more open canopy is important because it permits the growth of shrubs providing food and *M*. *avellanarius* can move in 2–5 m height on branches avoiding the forest floor [30]. In the sense of dimensional reduction it is therefore necessary to rely more on the ecological understanding of a species than purely on statistical functions [2]. The problem with high-detailed landscape data is that it is rarely area-covering or available on a larger scale. Therefore, we assessed if the model based on low detail is sufficient for conservation efforts. The results showed that the regional models covered almost the same (96% overlap) areas with a moderate to optimal habitat suitability. However, the lower overlap of optimal areas indicated that different model variables influence the determination of optimal habitats. Therefore, we concluded that low-detailed landscape models are valuable tools for large scale assessment of species distribution or prevalence modelling [64]. However, for effective regional species conservation e.g. selection of sites for the construction of new wind turbines high-detailed regional models must be used as the determination of optimal habitats is more realistic.

### Overlap of optimal habitat and wind energy precedence areas. A serious threat?

The different modeling approaches produced different overlaps with optimally suitable habitat for *M*. *avellanarius*. When analyzing habitat suitability in landscape planning the quality of species presence/absence data, sample size and included detail in the landscape data must be considered and evaluated. Landscape alterations are a serious threat to *M*. *avellanarius* [41, 63]. This species is sensitive to habitat loss, but also and maybe to a higher degree to habitat fragmentation due to e.g. road building or urban expansion [41, 63]. Due to its way of locomotion even newly installed or broadened forest tracks in the course of construction events might fragment suitable breeding or foraging habitats [30, 31]. These gaps might be crossed during migration as it is known that *M*. *avellanarius* can cross spaces of up to 100 m on the ground [31, 65]. However, during breeding and foraging the ground is avoided and therefore these gaps fragment potential habitat [57]. Even though the overlap of suitable habitats and precedence areas for wind turbines can be considered as low on a landscape-scale the fragmentation effect in optimal habitats might be substantial. The loss of 6% of optimal habitat can pose a serious threat on a regional scale and might lead to the extinction of local populations. The precedence areas were designated based on expert opinions but this study clearly demonstrated the necessity to include habitat suitability maps for endangered species in those considerations [66–71]. Even after designation of precedence areas high-detailed habitat suitability models can be used to choose less suitable habitat within a precedence area. This is, however, only possible when models use high-detailed site-specific data with ecological relevance for the study species.

### Conclusions

To increase sample size and model accuracy all available data sets for a given species should be merged including voluntarily surveyed data sets. These mostly include null samples that further improve model quality. Depending on the aim of the study e.g. assessment of large scale distribution low-detailed, freely available data sources as explanatory variables might be sufficient. For detailed regional conservation efforts high-detailed regional models should be used as the assessment of optimal habitats is more realistic. Modelers should be aware of the aim of their models and choose species and environmental data with the according level of detail.
